# Surface Electromyography Reveals Middle Deltoid as the Functionally Dominant Shoulder Muscle After Reverse Total Shoulder Arthroplasty

**DOI:** 10.7759/cureus.80229

**Published:** 2025-03-07

**Authors:** Alexander Pietroski, Yang Zhou, Johnny Kasto, Chimdindu Obinero, Kai Zhu, Mahdi Mazeh, Chaoyang Chen, Stephanie Muh

**Affiliations:** 1 Department of Orthopaedic Surgery, Henry Ford Health System, Detroit, USA; 2 Department of Biomedical Engineering, Wayne State University, Detroit, USA

**Keywords:** deltoid muscle, electromyography, muscle activation, reverse total shoulder arthroplasty, shoulder

## Abstract

Background: Reverse total shoulder arthroplasty (RSA) increases deltoid muscle fiber recruitment and tension to compensate for deficient rotator cuff activity; however, it is unclear whether the anterior or middle deltoid becomes dominant and how the muscle activation profile changes postoperatively. Using minimally invasive electromyography, this study evaluated the activity of the deltoid and surrounding muscles during shoulder motion to assess muscle activation changes post-RSA.

Methods: In this observational study, we assessed change in preoperative to postoperative shoulder muscle activation in 10 patients over six months. Muscle activation was measured using eight surface electrodes. Activation of the anterior, middle, and posterior deltoid, along with surrounding muscles, was recorded and quantified during shoulder abduction, flexion, and external and internal rotation. One-way analysis of variance was used to identify significant differences in activation and time or speed. The least significant difference post hoc test was used to determine specific differences in muscle activation at subsequent time points.

Results: RSA shoulders at six months postoperatively showed a significant increase in activity of the middle deltoid predominantly. Middle deltoid activation increased during abduction (p<0.001), flexion (p=0.008), external (p<0.001), and internal (p<0.001) rotation.

Conclusions: Our study highlights the middle deltoid as the primary contributor to rotator cuff function in reverse shoulder arthroplasty (RSA), characterized by quantitative activation, significant involvement in all shoulder motions, and increased activation over time. These findings could inform future RSA designs to enhance deltoid wrapping, maximizing strength and efficiency.

## Introduction

A reverse total shoulder arthroplasty (RSA) is indicated for patients with rotator cuff arthropathy, a disease characterized by both glenohumeral arthritis and rotator cuff insufficiency. The RSA prosthesis defies natural shoulder joint anatomy by converting the humeral head into a socket and the glenoid into a half sphere, resulting in a medialized center of rotation and a lengthened humerus [1,2]. This design alters the biomechanics of the shoulder, increasing deltoid muscle fiber recruitment and, ultimately, shoulder strength, granting superior stability and control compared to a conventional total shoulder arthroplasty [1]. Over the course of recovery, the rotator cuff and supporting muscles, particularly the deltoid, adapt to the shoulder’s altered biomechanics, having a significant impact on muscle activity, functional outcomes, and range of motion [3-5]. Muscle activity or adaptation can be assessed through muscle tests, invasively using small needle electrodes or with surface electrodes adhered to the skin. Surface electromyography (sEMG) has recently been shown to be a valid and noninvasive tool for quantifying individual muscle activation in the shoulder and has been used extensively in clinical diagnostic and rehabilitation settings [3,6-9]. 

Previous research consistently demonstrates that the deltoid is the dominant muscle post-RSA. At two-year follow-up, sEMG revealed significantly increased anterior and middle deltoid activation during abduction and flexion, while the posterior deltoid dominated during extension and external rotation [4]. 

The purpose of this study was to evaluate shoulder muscle activity and adaptation preoperatively and postoperatively using sEMG during arm forward flexion, abduction, and internal and external rotation. Furthermore, we aimed to determine the dominant deltoid head involved in each of the four motions as defined by quantitative activation (root mean square(RMS)), level of significant involvement in all four shoulder motions, and increased activation at all subsequent postoperative time points. We hypothesized that the middle deltoid would be the dominant muscle involved in forward flexion, abduction, and internal rotation, while the posterior deltoid would dominate during external rotation as described by sEMG. We provided data longitudinally at six weeks post-RSA (6w-RSA), three months post-RSA (3m-RSA), and six months post-RSA (6m-RSA) of the anterior, middle, and posterior deltoid, supraspinatus, infraspinatus, teres minor, upper trapezius, and lower trapezius to describe early muscle adaptations. 

This article was previously presented as a meeting abstract at the 2023 International Congress on Shoulder and Elbow Surgery (ICSES) on September 5th, 2023, and the 2021 European Society for Surgery of the Shoulder and Elbow (SECEC) Annual Congress on September 8th, 2021. 

This article was also previously posted to the Research Square preprint server on July 12th, 2021.

## Materials and methods

Patient evaluation 

Institutional review board approval from the Henry Ford Health System (IRB #11893) was obtained prior to data collection or analysis. From May 2019 to November 2019, ten subjects were recruited and consented. Indications for RSA surgery included degenerative glenohumeral osteoarthritis with a concomitant large rotator cuff tear. All subjects undergoing elective RSA were included unless they were to receive revision RSA surgery, RSA for a fracture, or declined to participate in the study. All subjects underwent surgery by the same surgeon at a single institution. All RSAs were performed on patients placed in the beach chair position through a deltopectoral approach. All components were implanted according to the manufacturer’s instructions (Equinoxe Reverse System, Exactech, Inc., Gainesville, FL). The prosthesis design included a medialized glenoid component with a lateralized humeral stem. 

Assessment of sEMG activity 

**Figure 1:**
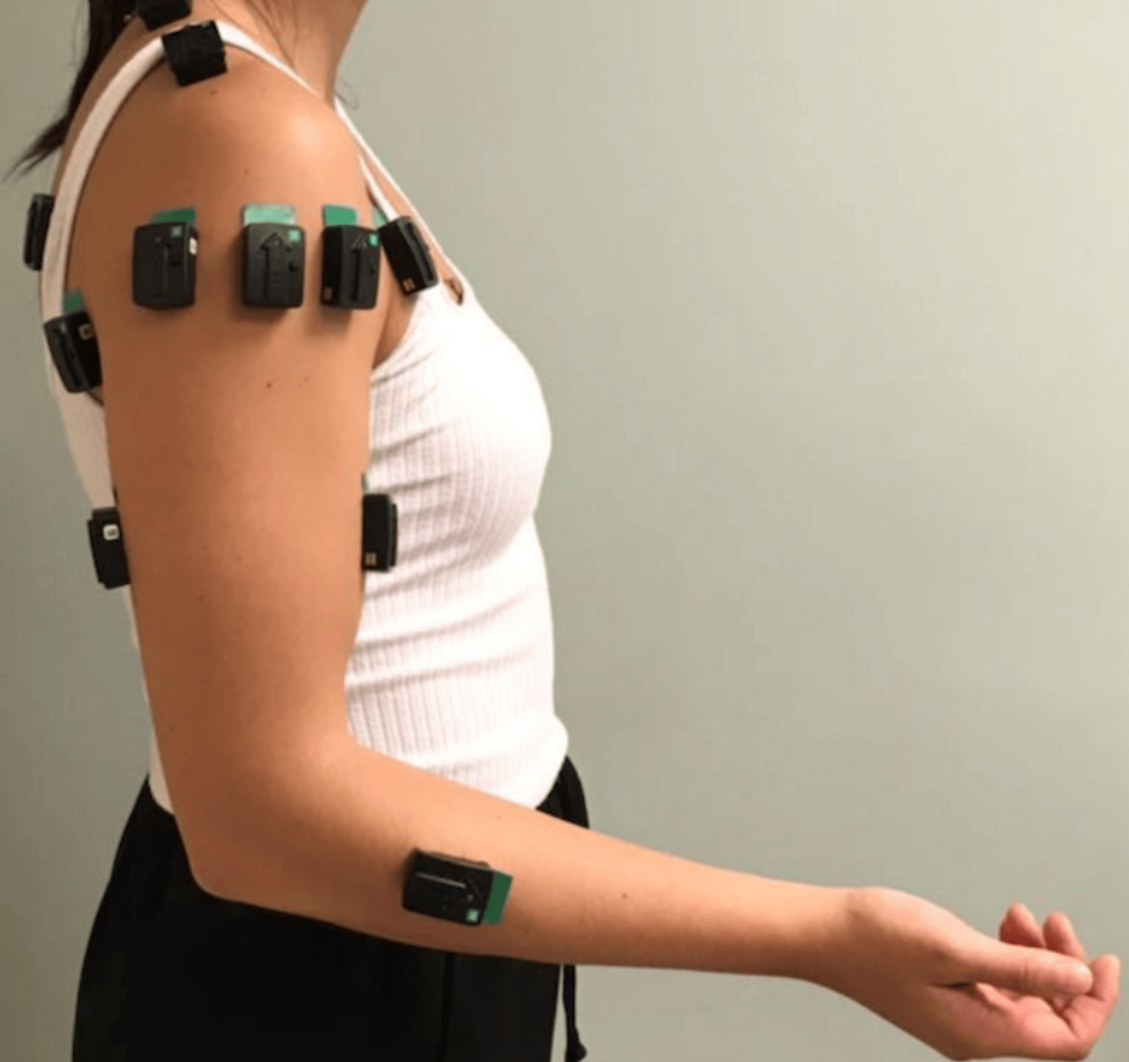
Sample photo sensors.

Patients were subsequently scheduled for a preoperative appointment before surgery to assess baseline non-RSA shoulder muscle activation using sEMG. For each of the ten subjects, eight wireless three-dimensional bluetooth sEMG sensors linked to a computerized analysis system (Model Trigno Avanti Platform, DELSYS Inc., Natick, MA) were used to measure muscle activation of eight shoulder muscles of the affected arm (Figure 1 and Table 1): anterior deltoid (Channel (Ch1)), middle deltoid (Ch2), posterior deltoid (Ch3), supraspinatus (Ch4), infraspinatus (Ch5), teres minor (Ch6), upper trapezius (Ch7), and lower trapezius (Ch8). To minimize signal disruption, sEMG sensors were placed on the central point of muscle fibers and oriented toward the muscle origin after the skin region was rubbed with an alcohol pad. Subjects performed four dynamic motions in series (abduction, forward flexion in the scapular plane, external rotation, and internal rotation) at three different speeds (slow, medium, and fast). Before each sEMG measurement, the patient was shown the motion to be completed. Patients who did not complete satisfactory motions for a given exercise were asked to repeat that trial. If they were unable to complete it due to limited shoulder ability, that trial was excluded. At each speed, each of the four shoulder motions was completed three times with three seconds of rest between each repetition. Patients were given approximately one minute of rest between each activity to limit fatigue and were instructed to request more rest time if needed. The same motions were repeated and recorded at pre-op, 6w-RSA, 3m-RSA, and 6m-RSA. 

**Table 1:** Electrode placements.

Muscle	Placement of electrode
Anterior deltoid	Middle point with anterior line of the muscle width and length.
Middle deltoid	Middle point with the middle line of the muscle width and length.
Posterior deltoid	Middle point with posterior line of the muscle width and length.
Upper trapezius	Midpoint between acromion and C7 vertebral spine
Lower trapezius	T2-T3 directly inferior to upper trapezius
Supraspinatus	0.5 thumb widths superior to midpoint of scapular spine
Infraspinatus	1-2 thumb widths inferior to midpoint of scapular spine
Teres minor	1-2 thumb widths inferior and lateral to greater tubercle of humerus

All raw EMG data signals were sampled at 1.11 kHz and filtered via third order Butterworth filter at 20-350 Hz, which is the default setting on the Delsys EMG system (Delsys Inc., Natick, MA). The original data was stored in an EMGWorks (Delsys Inc., Natick, MA) specific file format and then exported in comma-separated values data format for data processing. The third-order Butterworth notch filter at 60, 180, and 300Hz was used to remove power line noise [10]. Analysis was used to determine the speed and degree of muscle activation (RMS) for each of the eight muscles during the four different shoulder movements using EMGWorks. The Delsys Trigno wireless EMG acquisition system (Delsys Inc., Natick, MA) and EMGWorks Acquisition software (version 4.7.6, Delsys Inc., Natick, MA) were used for EMG recording. The Delsys system was connected to a computer controlled by the EMGWorks software via a USB cable. The wireless sensors were attached to the skin using a specially designed double-sided adhesive tape. Each wireless sensor had four dry EMG electrodes to collect muscle activities during arm movements. Each sensor had an EMG sensor, an accelerometer, and a gyroscope. The shoulder motion speed was determined by measuring the angle from gyroscope readings and time reading from the accelerometer. Analyses were performed by a single researcher for consistency. MATLAB (The MathWorks Inc, Natick, MA) was used for the EMG signal segmentation into datasets according to the designated time points along with the whole recording procedure. 

To normalize EMG values among subjects for analysis and mitigate signal variation throughout the course of a motion, the filtered EMG signal was measured from a point after motion initiation to a point prior to termination of the signal. This time frame was kept standard in each patient’s analysis. Although EMG activity is usually normalized through reference to maximum voluntary contraction, the severe RC deficiency in RSA prohibited this. One patient declined to participate in 6w-RSA testing, and two patients were unable to complete 3m-RSA testing due to the constraints of the COVID-19 pandemic. 

EMG data analysis 

The root mean square (RMS) of EMG signal amplitude was analyzed to determine the activation of the muscle [11,12]. The RMS is the most common method used for muscle activation and EMG pattern recognition research [13]. The mathematical expression of RMS is as the following equation: 

$RMS = \sqrt{\frac{1}{N} \sum_{i=1}^{N} V(i)^2}$

Statistical analysis 

One-way analysis of variance and general linear model univariate with Post Hoc least significant difference (LSD) was used to identify any significant difference in muscle activation and time or muscle activation and speed. LSD post hoc test was used to determine specific differences in muscle activation between time points (preoperative, 6w-RSA, 3m-RSA, and 6m-RSA). All analyses were performed using a level of significance at α=0.05. SPSS software was used for all statistical analyses (version 26, IBM Corp, Armonk, NY). 

## Results

The patient population studied was 30% male and 70% female, aged 71.6±4.543 years on average. Table 2 displays preoperative and postoperative sEMG muscle activation outputs. Six-month post-RSA shoulders showed a significant increase in middle deltoid activity predominantly, as well as the anterior deltoid, upper trapezius, and posterior deltoid across several shoulder motions when compared to pre-RSA shoulders (univariate post hoc least significant difference, p<0.05). There were no significant differences in any muscle activation between slow, medium, and fast speeds for any motion except the middle deltoid forward flexion at cumulative time points (slow to fast motion speed, p=0.0492). 

**Table 2:** Electromyograph amplitudes by muscle group and motion at preoperative and 6 month postoperative timepoints. EMG: electromyography; Pre-RSA: preoperative-reverse total shoulder arthroplasty ; 6m-RSA, 6-months postoperative-reverse total shoulder arthroplasty; RMS: root mean square; V: Volts; SE: standard error.

Motion	EMG amplitude, RMS (V) ± SE				
	Anterior deltoid	Middle deltoid	Posterior deltoid	Upper trapezius	Lower trapezius
Abduction Pre-RSA	0.812 ± 0.2	0.702 ± 0.2	0.645 ± 0.2	0.520 ± 0.1	0.293 ± 0.2
Abduction 6m-RSA	1.581 ± 0.2	1.726 ± 0.2	0.812 ± 0.2	1.159 ± 0.1	0.710 ± 0.2
Abduction p-value	0.0007	0.0014	0.0216	0.0000	0.0260
Forward flexion Pre-RSA	1.900 ± 1.4	0.533 ± 0.2	1.647 ± 0.8	0.303 ± 0.1	0.306 ± 0.2
Forward flexion 6m-RSA	1.527 ± 1.5	1.566 ± 0.2	0.916 ± 0.9	0.844 ± 0.1	0.711 ± 0.2
Forward flexion p-value	0.3444	0.0000	0.2614	0.0002	0.03134
External rotation Pre-RSA	0.603 ± 0.4	0.289 ± 0.2	1.061 ± 0.6	0.214 ± 0.3	0.429 ± 0.2
External rotation 6m-RSA	1.931 ± 0.5	0.880 ± 0.2	1.983 ± 0.7	0.448 ± 0.3	1.051 ± 0.2
External rotation p-value	0.0818	0.0000	0.0437	0.7510	0.0194
Internal rotation Pre-RSA	0.290 ± 0.3	0.262 ± 0.1	0.549 ± 0.3	0.225 ± 0.2	0.668 ± 0.3
Internal rotation 6m-RSA	0.799 ± 0.4	0.856 ± 0.1	0.904 ± 0.4	0.799 ± 0.2	0.713 ± 0.2
Internal rotation p-value	0.0435	0.0000	0.0134	0.0045	0.5426

**Figure 2:**
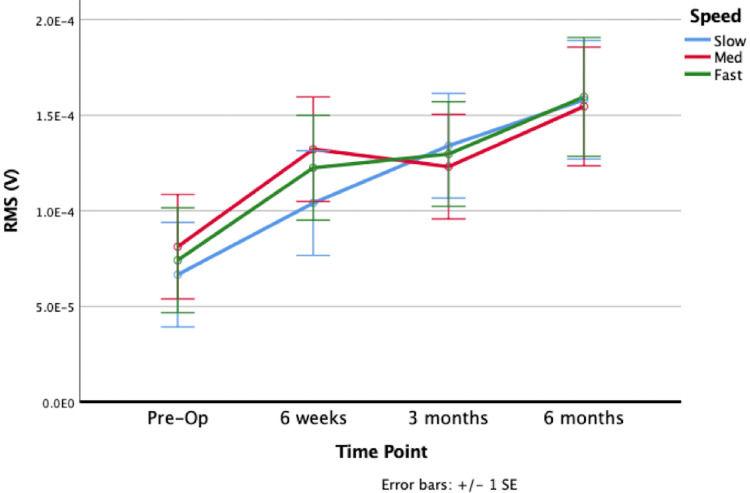
Anterior deltoid sEMG activation during abduction at pre-RSA, 6-week RSA, 3-month RSA, and 6-month RSA. EMG, electromyography; Pre-op, preoperative; RMS, root mean square; RSA, reverse total shoulder arthroplasty; SE, standard error.

**Figure 3:**
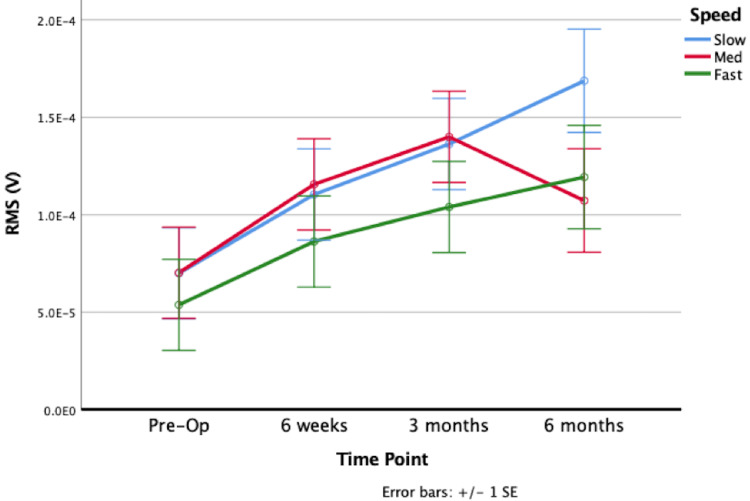
Middle deltoid sEMG activation during abduction at pre-RSA, 6 week RSA, 3 month RSA, and 6 month RSA. Abbreviations: EMG, electromyography; Pre-op, preoperative; RMS, root mean square; RSA, reverse total shoulder arthroplasty; SE, standard error.

**Figure 4:**
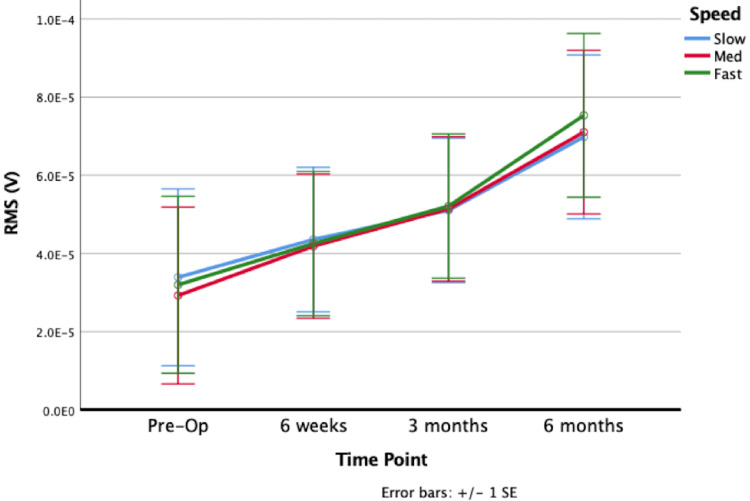
Lower trapezius sEMG activation during abduction at pre-RSA, 6 week RSA, 3 month RSA, and 6 month RSA. Abbreviations: EMG, electromyography; Pre-op, preoperative; RMS, root mean square; RSA, reverse total shoulder arthroplasty; SE, standard error.

The dominant muscles in pre-RSA abduction were the anterior and middle deltoid. Compared to pre-RSA, 6m-RSA shoulder abduction displayed significantly increased sEMG activity of the anterior deltoid (p=0.0007; Figure 2), middle deltoid (p=0.0014; Figure 3), posterior deltoid (p=0.0216), upper trapezius (p=0.0000) and lower trapezius (p=0.0260; Figure 4); however, the middle deltoid showed both the greatest increase in quantitative RMS activation (72.6%), and increase in activation (139%). All five muscles showed increased activation at nearly all subsequent postoperative time points (from 6w-RSA to 3m-RSA and 3m-RSA to 6m-RSA), although this increase in activation was not always statistically significant. The supraspinatus (p=0.5262), infraspinatus (p=0.5195), and teres minor (p=0.4352) did not show a significant increase in muscle activation during abduction from pre-RSA to 6m-RSA. 

In pre-RSA forward flexion, the dominant muscles were the teres minor and infraspinatus. Post-RSA forward flexion, the middle deltoid emerged as the dominant muscle, exhibiting the highest increase in quantitative RMS activation at 56.6% and an overall activation increase of 194%. During forward flexion, the middle deltoid (p=0.0000) and upper trapezius (p=0.0000) showed significantly increased muscle activation comparing 6m-RSA shoulders to pre-RSA shoulders, and both muscle fibers trended upward in activation at nearly all subsequent postoperative time points. Although the anterior deltoid showed a significant increase in activation from 6w-RSA to 3m-RSA (p=0.0250), the anterior and posterior deltoid had a quantitative decrease in sEMG activation from pre-RSA to 6m-RSA. The supraspinatus (p=0.7836), infraspinatus (p=0.4153), teres minor (p=0.7053), and lower trapezius (p=0.3134) did not show a significant increase in muscle activation during forward flexion. 

There were no specific predominant muscles in pre-RSA external rotation. Post-RSA, the anterior and posterior deltoid showed the greatest quantitative sEMG activation during external rotation. The middle deltoid (p=0.0000), posterior deltoid (p=0.0437), and lower trapezius (p=0.0194) showed significantly increased sEMG activity in 6m-RSA shoulders compared to pre-RSA shoulders, and all three muscle fibers showed increased activation at subsequent postoperative time points. The anterior deltoid (p=0.0818), supraspinatus (p=0.7877), infraspinatus ( p=0.1413), teres minor (p=0.5031), and upper trapezius (p=0.7510) did not show a significant increase in muscle activation during external rotation from pre-RSA to 6m-RSA. 

There were no specific predominant muscles in pre-RSA internal rotation. Postoperatively, the middle and posterior deltoid showed the greatest quantitative sEMG activation during internal rotation. The anterior deltoid ( p=0.0435), middle deltoid (p=0.0000), posterior deltoid (p=0.0134), supraspinatus (p=0.0051), teres minor (p=0.0503) and upper trapezius (p=0.0045) showed significantly increased sEMG activity in 6m-RSA shoulders compared to pre-RSA shoulders. All six muscle fibers showed an overall increased activation at consecutive postoperative time points. The infraspinatus (p=0.7085) and lower trapezius (p=0.5244) did not show a significant increase in muscle activation during internal rotation from preoperatively to 6m-RSA. 

## Discussion

Previous literature has demonstrated that the deltoid is the dominant muscle in shoulder motion post-RSA [3-5,14]. Modern RSA prosthesis lengthens the deltoid, increasing muscle fiber recruitment to lend strength and assist in dynamically stabilizing the shoulder regardless of an insufficient rotator cuff [15]. However, it is unclear which specific deltoid head adapts to become the dominant shoulder muscle across multiple movements and how those adaptations occur in the early months after surgery. At two years post-RSA, there was a significant increase in anterior and middle deltoid activation during abduction, and flexion was detected using sEMG. The posterior deltoid showed predominant sEMG activation during shoulder extension and external rotation. The group also noticed a significant loss of deltoid efficiency through increased fatiguability [4], which may contribute to long-term degeneration and fatty infiltration [16]. Anterior and posterior deltoid sEMG quantitative activation has also demonstrated an ability to predict postoperative range of motion, American Shoulder and Elbow Surgeons score, and Constant score, reaffirming the deltoid as the most important factor in postoperative RSA outcomes [3]. This has implications for both the surgeon and the patient in deciding which part of the deltoid to disrupt during an open rotator cuff repair approach [17,18]. Findings could also educate future RSA prosthesis design choices to maximize deltoid wrapping to increase efficiency, strength, stability, and range of motion of the shoulder [19,20]. 

This study demonstrates through sEMG analysis that the middle deltoid adapts to become the defining muscle of shoulder activity over the anterior deltoid in 6m-RSA shoulders. We define dominance as achieving the highest quantitative activation (RMS) level of significant involvement in all four shoulder motions and increasing RMS at all subsequent postoperative time points. The middle deltoid achieved the highest RMS, was involved in the most shoulder movements, and trended towards significance across the first six months postoperative. This is in contrast to the anterior deltoid, which showed a lesser quantitative activation, did not show significant increases in activation during forward flexion or external rotation post-RSA, and showed a less significant increase in activation at subsequent post-RSA time points compared to the middle deltoid. These findings address the current controversy of whether the middle or anterior head of the deltoid dominates postoperatively in RSA shoulders. 

The middle deltoid dominating shoulder function post-RSA has significant implications for patients who have had previous surgeries on the same shoulder. Any lateral approach to the shoulder, such as seen in open rotator cuff repairs, may disrupt the integrity of the middle deltoid [21-24]. This may result in sub-optimal outcomes after conversion to an RSA. Surgeons may want to consider alternative approaches to the rotator cuff if a future RSA is likely. Furthermore, there is always a chance for complete or partial axillary or suprascapular nerve denervation when using a lateral shoulder approach [25-27] in addition to denervation that may occur during the RSA procedure itself [17,28]. Careful consideration should be given to maintain the integrity of the middle deltoid and associated nerves to preserve postoperative functionality. 

These findings may inform future RSA prosthesis design by directing manufacturers to target the middle deltoid to enhance the deltoid wrap. The deltoid wrap is the concept that the deltoid forms a lever arm by using the lateral aspect of the proximal head of the humerus as a fulcrum to increase the distance to its insertion [2]. The deep surface of the deltoid, under greater tension, wraps over the head of the humerus, providing a compressive downward and medial force lending greater stability. If biomechanically optimized, RSA prosthesis should lend greater leverage and efficiency. Currently, RSA shoulders work harder to achieve a lower range of motion when compared to a healthy shoulder [5]. This alludes to a more optimal RSA design building on the current medialized glenosphere and lateralized humerus designs, which should target the middle deltoid to enhance stability, range of motion, and strength maximally. 

This analysis demonstrates that RSA shoulders have a significantly different pattern of muscle activation at six months postoperatively when compared to pre-RSA. Two years after surgery, Pegreffi et al. EMG showed activation of the anterior and lateral deltoid significantly decreased in patients who underwent reverse shoulder prosthesis compared to the contralateral shoulder despite low reported pain levels [29]. Our results showed a dominant and integral role of the middle deltoid with supporting activation of the anterior and posterior deltoid in reverse shoulder prosthesis patients. These findings suggest that a minimum of two years of follow-up is required to fully evaluate clinical outcomes and address the biomechanical and functional limitations of this procedure. It may be the case that deltoid activation peaks sometime at six months, only to decrease below the patient’s contralateral arm activation. This finding may be attributed to increased stress, decreased efficiency, and increased wear on the deltoid. Further studies are encouraged to evaluate long-term deltoid habituation to RSA implantation along with objective measures of outcomes, such as range of motion and visual analog scale. 

Li et al. demonstrated the accuracy and utility of preoperative EMG activity as a predictor of RSA outcome [3]. Pre-RSA patients with increased middle deltoid and upper trapezius activity showed greater post-RSA shoulder strength. Furthermore, pre-RSA anterior and middle deltoid EMG activity correlated with postoperative range of motion for abduction and flexion, and posterior deltoid EMG activity correlated with postoperative range of motion for external rotation. These findings are supported by our results, which show the most significant quantitative increase in RMS activity for the middle deltoid and upper trapezius on RSA shoulders. Furthermore, our results show the middle and anterior deltoid muscles predominate in quantitative EMG activation during abduction and forward flexion, and the posterior deltoid predominates quantitatively in external rotation. 

Our sEMG also revealed significantly increased activation of the upper trapezius during abduction, forward flexion, and internal rotation. This finding supports previous literature demonstrating increased activation at one and two-year follow-ups with abduction and forward flexion but not internal rotation [3,5]. We hypothesize that the upper and lower trapezius may act as an involuntary stabilizing force during deltoid contraction to compensate for new biomechanics. However, high activation of the upper trapezius may also be due to voluntary patient accommodation shrugging motion to compensate for weakness, stiffness, or pain [5]. 

This study is not without limitations. Wherever possible, we sought to mitigate experimental variability with a single researcher preparing and placing the sEMG sensors for all patients at all time points and that same researcher instructing patients through the shoulder motion. We also repeated or discarded trials that were not satisfactory. Even so, EMG data can show variability, especially within a small sample size. Several patients were unable to complete the three-month EMG testing or experienced delays in the six-month testing due to COVID-19 restrictions. However, since the endpoint for post-RSA shoulder recovery is widely considered to be between six months and one year [30], focusing on the changes in muscle activation from the preoperative period to six months or later postoperatively should provide a comprehensive clinical understanding of the activation changes induced by RSA.

## Conclusions

This study suggests the middle deltoid is the dominant muscle during flexion, abduction, internal rotation, and external rotation, with significant co-activation of the anterior deltoid during abduction and posterior deltoid during internal rotation in the post-RSA shoulder. Over the course of six months, the middle deltoid adapts to altered shoulder biomechanics and displays greater quantitative activation (RMS), level of significant involvement in all four shoulder motions, and increased activation trending toward significance at all subsequent postoperative time points. These findings may guide RSA prosthesis design to optimize deltoid wrapping around the middle deltoid, maximizing strength and efficiency. Furthermore, surgeons should be cognizant of lateral shoulder approaches, which disrupt the middle deltoid, and possible denervation, which may affect RSA outcomes. 
